# Non-conventional dosing of oral anticancer agents in oncology and malignant haematology: a systematic review protocol

**DOI:** 10.1186/s13643-017-0636-y

**Published:** 2017-12-06

**Authors:** Faouzi Djebbari, Nicola Stoner, Verna Lavender

**Affiliations:** 1 NIHR Oxford Biomedical Research Centre, Oxford, OX3 7LE UK; 2 Oxford Cancer and Haematology Centre, Churchill Hospital, Oxford University Hospitals NHS Foundation Trust, Oxford, OX3 7LE UK; 3 School of Chemistry, Food and Pharmacy, University of Reading, Reading, UK; 4 Faculty of Health and Life Sciences, Oxford Brookes University, Jack Straw’s Lane, Marston Road, Oxford, OX3 0FL UK

**Keywords:** Cancer, Oncology, Haematology, Anticancer, Antineoplastic, Oral drug, Non-conventional dose, Non-standard dose, Systematic review

## Abstract

**Background:**

Recent advances in cancer therapeutics have resulted in significantly improved overall survival and progression-free survival for patients. Targeted oral systemic anticancer therapies (SACT) offer a range of treatment approaches that differ from traditional cytotoxic chemotherapy: non-cytotoxic oral SACT target malignant disease continuously, have less broad and more favourable safety profiles, which can improve patients’ quality of life (QoL). Toxicities associated with daily oral SACT administration can, however, result in non-adherence and a reduced QoL. Non-conventional dosing of oral SACT, where unlicensed doses/schedules of drugs are prescribed, is one approach increasingly adopted by clinicians to reduce toxicities and subsequent non-adherence and to improve QoL. Guidance governing this practice is, however, limited. This systematic review aims to identify evidence about prescribing practices of, and outcomes from, non-conventional dosing of oral SACT in oncology and malignant haematology.

**Methods:**

A search using the following electronic databases will be conducted: Ovid MEDLINE, Ovid EMBASE, Cumulative Index to Nursing and Allied Health Literature (CINAHL) and Cochrane Registry of Controlled Trials. Studies will be selected based on predefined inclusion/exclusion criteria. Critical appraisal will be conducted to identify potential biases, strengths and limitations of included studies. Extracted data will be tabulated to sort and summarise key findings. An initial literature search indicated that studies reporting non-standard dosing of oral SACT intervention studies are diverse and heterogeneous in study design. Extracted data will, therefore, be tabulated, and together with a narrative synthesis of integrated key findings, will be presented and discussed in reference to the strengths and weaknesses of the evidence base. If sufficient stratified data is available (e.g. age group, tumour type, disease stage) or intervention (drug, dosing schedule), sub-group analysis will be conducted to inform prescribing practice.

**Discussion:**

This review will identify relevant literature on the topic to inform prescribers working in oncology and malignant haematology. It will also analyse any evidence of the following outcomes: toxicity, treatment adherence and/or QoL outcomes for patients receiving non-standard doses of oral SACT. Limitations in the evidence base may arise from variability in both the type and quality of studies reviewed.

**Systematic Review Registration:**

PROSPERO CRD42017076195.

## Background

Major advances in drug development have revolutionised cancer treatment over the last decade [1]. Oral systemic anticancer therapies (SACT) refer to orally administered drugs with an antineoplastic indication and include traditional cytotoxics (e.g. cyclophosphamide, capecitabine, busulfan), small molecule targeted agents (e.g. imatinib, pazopanib, sunitinib), immunomodulatory drugs (e.g. thalidomide, lenalidomide, pomalidomide), and hormone modulators (e.g. tamoxifen, enzalutamide, abiraterone) [2]. Unlike injectable cytotoxic chemotherapy, where therapeutic doses are administered at specific days on a cyclical basis, oral SACT (particularly targeted therapies) are given as continuous, often daily treatments, with a possible scheduled break depending on the licensed dose of the drug in question [2].

Chemotherapy dose rounding and banding is a well-established current practice in the UK, which helps to maintain consistent prescribing of oral SACT throughout a patient’s treatment journey. However, daily oral administration requires a high level of adherence to maximise clinical benefit, which patients can find challenging [3]. Experiences from both clinical studies and current practice suggest that non-adherence to conventional dosing of these drugs can be directly attributed to incidents and severity of side effects [4]. Non-adherence due to side effects can negatively affect efficacy outcomes from treatment. In parallel, intolerable side effects can also adversely affect patients’ QoL for the duration of their treatment and beyond (if side effects are irreversible). Toxicities and related poor QoL outcomes, measured by instruments such as the European Organisation for Research and Treatment of Cancer (EORTC) QLQ-C30 questionnaire [5], can ultimately lead to dose reductions, treatment interruption or discontinuation [6].

Drug efficacy is measured by specific disease-response assessment criteria, such as response evaluation criteria for solid tumours (RECIST) [7], and International Myeloma Working Group (IMWG) criteria for multiple myeloma [8]. To ensure safe and effective treatment, it is necessary for prescribers (medical and non-medical) to exercise informed decisions when practice does not follow standard dosing. Non-conventional dosing of oral SACT, where unlicensed doses/schedules are prescribed, is increasingly being used to reduce adverse outcomes described above.

There is very limited guidance on the use of non-conventional dosing of oral SACT. For instance, there is no guidance in the UK to recommend or govern their non-conventional prescribing for the treatment of multiple myeloma. Therefore, this systematic review aims to identify evidence about non-standard dosing of these agents in oncology and malignant haematology to inform prescribing practices. A secondary aim of this review is to analyse any evidence of the following outcomes: toxicity, treatment adherence and/or QoL outcomes for patients receiving non-standard doses of oral SACT to inform research questions evaluating the feasibility of non-standard prescribing strategies for these therapies.

## Methods

### Study design

The proposed systematic review will be undertaken between March 2017 and March 2018, following an a priori protocol, as described herein. The protocol for the review was designed in accordance with the Preferred Reporting Items for Systematic Review and Meta-Analysis Protocols (PRISMA-P) guidelines [9], which has been registered with the PROSPERO database (CRD42017076195). The review will meet criteria for a systematic review described by Grant and colleagues [10]. It will systematically search for evidence in an exhaustive and comprehensive manner without time limitation or scoping constraints, and will apply predefined inclusion and exclusion criteria. Quality appraisal of studies will be integral to the review’s inclusion and exclusion process. The review will also conduct sub-group analyses if findings allow. Any methodological changes from the protocol to the final review will be documented in the subsequent manuscript and submitted for publication.

### Eligibility criteria

Studies will be included if they meet the following criteria:

- Studies of malignant disease
- Studies of patients aged ≥18 years
- Studies of oral SACT with non-conventional dosing
- Studies examining the prescribing practices using unlicensed (non-standard) doses or schedules of oral SACT
- Meta-analysis
- Late phase clinical trials
- Cohort studies
- Cross-sectional studies
- Retrospective studies
- Observational studies
- Case-control studies
- Case-reports
- MHRA: reports, legislative documents

The following publications will be excluded:

- Studies of parenteral SACT (e.g. IM, IV, SC, IT)
- Studies of oral SACT where non-conventional dosing has been used, but cannot be extracted independently of other reported data
- Studies comparing different licensed doses of oral SACT for the same antineoplastic indication
- New standard dose-finding studies
- Animal studies
- Early phase clinical trials
- Pharmacokinetic studies
- Narrative reviews
- Opinion papers
- Education papers
- Commentaries
- Editorials
- Conference abstracts

To reduce the risk of bias generated from reviewing interpretative secondary analysis, systematic reviews other than meta-analyses will also be excluded. Systematic reviews will be used to source relevant primary research from reference lists as part of the search expansion process.

### Search strategy

The PROSPERO database was searched to confirm that no systematic reviews were/are being conducted on this subject. The following electronic resources will be searched: Ovid MEDLINE, Ovid EMBASE, Cochrane Registry of Controlled Trials, and Cumulative Index to Nursing and Allied Health Literature (CINHAL). The search expansion might also include searching the following grey literature sources: Medicines and Healthcare Products Regulatory Agency (MHRA) website, and Clinicaltrials.gov website.

A preliminary systematic search strategy will be tested in order to achieve sufficient specificity while maintaining high sensitivity. Boolean operators AND, and OR will be used, as well as truncation (*). Searches will include title and abstract where possible, and will be restricted to English language only. There will be no date restriction.

Search strategy contains 3 components (drug names, relevant MESH terms for antineoplastic agents, and all terms describing non-conventional dosing).

- Component one:

All 77 oral SACT listed in the British National Formulary (BNF) will be searched as MESH terms [2]. If a drug does not have a MESH term on any or all of the databases, it will be searched for as a keyword. Search terms for cytotoxic agents are: bexarotene, busulfan, capecitabine, chlorambucil, cyclophosphamide, estramustine, etoposide, hydroxycarbamide, melphalan, mercaptopurine, lomustine, mitotane, procarbazine, tegafur, temozolomide, tioguanine, topotecan, treosulfan, tretinoin, trifluridine, vinorelbine, idarubicin, and methotrexate. Search terms for small molecule targeted therapies are: afatinib, axitinib, bosutinib, cabozantinib, cobimetinib, crizotinib, ceritinib, dabrafenib, dasatinib, erlotinib, everolimus, gefitinib, ibrutinib, idelalisib, imatinib, lapatinib, nilotinib, nintedanib, olaparib, osimertinib, panobinostat, pazopanib, ponatinib, regorafenib, ruxolitinib, sorafenib, sunitinib, temsirolimus, trametinib, vandetanib, vemurafenib, vismodegib, lenvatinib, palbociclib, venetoclax, and ixazomib. Search terms for immunomodulatory drugs are: thalidomide, lenalidomide, and pomalidomide. Search terms for hormone modulators are: anastrozole, exemestane, letrozole, tamoxifen, toremifene, abiraterone, bicalutamide, cyproterone, enzalutamide, flutamide, medroxyprogesterone, megestrol, norethisterone, diethylstilbestrol, and ethinylestradiol.

- Component two:

MEDLINE MESH: antineoplastic agents (administration and dosage), antineoplastic agents (therapeutic use), and drug administration schedule. EMBASE MESH: antineoplastic agent (drug administration), antineoplastic agent (drug therapy), antineoplastic agent (oral drug administration), antineoplastic agent (drug dose). CINAHL MESH: antineoplastic agents (administration and dosage), antineoplastic agents (therapeutic use), and drug administration schedule. Cochrane Registry of Controlled Trials keywords: drug administration, and antineoplastic agent.

- Component three:

The following terms will be used to search titles: reduc* or attenuat* or discontinu* or modif* or dosing or schedul* or strateg* or dosage* or intercalat* or alternat* or personali* or break or holiday* or cessation or interrup* or intermit* or escala* or prescrib* or optimis* or optimiz* or tailor* or individuali* or “non conventional*” or nonconventional* or “non standard*” or nonstandard* or “treatment free”.

Full database search strategies are shown in the following Tables: Table 1 (MEDLINE search strategy), Table 2 (EMBASE search strategy), Table 3 (CINAHL search strategy), and Table 4 (Cochrane Registry of Controlled Trials search strategy).

**Table 1:** MEDLINE search strategy

Search steps
1. Imatinib Mesylate/
2. bexarotene.mp.
3. Busulfan/
4. Capecitabine/
5. Chlorambucil/
6. Cyclophosphamide/
7. Estramustine/
8. Etoposide/
9. Hydroxyurea/ or hydroxycarbamide.mp.
10. Melphalan/
11. mercaptopurine.mp. or 6-Mercaptopurine/
12. Lomustine/
13. Mitotane/
14. Procarbazine/
15. Tegafur/
16. temozolomide.mp.
17. Thioguanine/
18. Topotecan/
19. treosulfan.mp.
20. Tretinoin/
21. Trifluridine/
22. vinorelbine.mp.
23. afatinib.mp.
24. axitinib.mp.
25. bosutinib.mp.
26. cabozantinib.mp.
27. cobimetinib.mp.
28. crizotinib.mp.
29. ceritinib.mp.
30. dabrafenib.mp.
31. Dasatinib/
32. Erlotinib Hydrochloride/
33. Everolimus/
34. gefitinib.mp.
35. ibrutinib.mp.
36. idelalisib.mp.
37. lapatinib.mp.
38. nilotinib.mp.
39. nintedanib.mp.
40. olaparib.mp.
41. osimertinib.mp.
42. panobinostat.mp.
43. pazopanib.mp.
44. ponatinib.mp.
45. regorafenib.mp.
46. ruxolitinib.mp.
47. sorafenib.mp.
48. sunitinib.mp.
49. temsirolimus.mp.
50. trametinib.mp.
51. vandetanib.mp.
52. vemurafenib.mp.
53. vismodegib.mp.
54. Thalidomide/
55. lenalidomide.mp.
56. pomalidomide.mp.
57. anastrozole.mp.
58. exemestane.mp.
59. letrozole.mp.
60. Tamoxifen/
61. Toremifene/
62. Abiraterone Acetate/
63. bicalutamide.mp.
64. Cyproterone/ or Cyproterone Acetate/
65. enzalutamide.mp.
66. Flutamide/
67. Medroxyprogesterone/ or Medroxyprogesterone Acetate/
68. Megestrol/ or Megestrol Acetate/
69. norethisterone.mp. or Norethindrone/
70. Diethylstilbestrol/
71. Ethinyl Estradiol/ or ethinylestradiol.mp.
72. Idarubicin/
73. Methotrexate/
74. Trifluridine/
75. lenvatinib.mp.
76. venetoclax.mp.
77. ixazomib.mp.
78. Palbociclib.mp.
79. 1 or 2 or 3 or 4 or 5 or 6 or 7 or 8 or 9 or 10 or 11 or 12 or 13 or 14 or 15 or 16 or 17 or 18 or 19 or 20 or 21 or 22 or 23 or 24 or 25 or 26 or 27 or 28 or 29 or 30 or 31 or 32 or 33 or 34 or 35 or 36 or 37 or 38 or 39 or 40 or 41 or 42 or 43 or 44 or 45 or 46 or 47 or 48 or 49 or 50 or 51 or 52 or 53 or 54 or 55 or 56 or 57 or 58 or 59 or 60 or 61 or 62 or 63 or 64 or 65 or 66 or 67 or 68 or 69 or 70 or 71 or 72 or 73 or 74 or 75 or 76 or 77 or 78
80. Antineoplastic Agents/ad [Administration & Dosage]
81. Antineoplastic Agents/tu [Therapeutic Use]
82. Drug Administration Schedule/
83. 80 or 81 or 82
84. (reduc* or attenuat* or discontinu* or modif* or dosing or schedul* or strateg* or dosage* or intercalat* or alternat* or personali* or break or holiday* or cessation or interrup* or intermit* or escala* or prescrib* or optimis* or optimiz* or tailor* or individuali* or "non conventional*" or nonconventional* or "non standard*" or nonstandard* or "treatment free").ab,ti.
85. (reduc* or attenuat* or discontinu* or modif* or dosing or schedul* or strateg* or dosage* or intercalat* or alternat* or personali* or break or holiday* or cessation or interrup* or intermit* or escala* or prescrib* or optimis* or optimiz* or tailor* or individuali* or "non conventional*" or nonconventional* or "non standard*" or nonstandard* or "treatment free").ti.
86. 79 and 83 and 84
87. 79 and 83 and 85

**Table 2:** EMBASE search strategy

Search steps
1. Imatinib Mesylate/
2. bexarotene.mp.
3. Busulfan/
4. Capecitabine/
5. Chlorambucil/
6. Cyclophosphamide/
7. Estramustine/
8. Etoposide/
9. Hydroxyurea/ or hydroxycarbamide.mp.
10. Melphalan/
11. mercaptopurine.mp. or 6-Mercaptopurine/
12. Lomustine/
13. Mitotane/
14. Procarbazine/
15. Tegafur/
16. temozolomide.mp.
17. Thioguanine/
18. Topotecan/
19. treosulfan.mp.
20. Tretinoin/
21. Trifluridine/
22. vinorelbine.mp.
23. afatinib.mp.
24. axitinib.mp.
25. bosutinib.mp.
26. cabozantinib.mp.
27. cobimetinib.mp.
28. crizotinib.mp.
29. ceritinib.mp.
30. dabrafenib.mp.
31. Dasatinib/
32. Erlotinib Hydrochloride/
33. Everolimus/
34. gefitinib.mp.
35. ibrutinib.mp.
36. idelalisib.mp.
37. lapatinib.mp.
38. nilotinib.mp.
39. nintedanib.mp.
40. olaparib.mp.
41. osimertinib.mp.
42. panobinostat.mp.
43. pazopanib.mp.
44. ponatinib.mp.
45. regorafenib.mp.
46. ruxolitinib.mp.
47. sorafenib.mp.
48. sunitinib.mp.
49. temsirolimus.mp.
50. trametinib.mp.
51. vandetanib.mp.
52. vemurafenib.mp.
53. vismodegib.mp.
54. Thalidomide/
55. lenalidomide.mp.
56. pomalidomide.mp.
57. anastrozole.mp.
58. exemestane.mp.
59. letrozole.mp.
60. Tamoxifen/
61. Toremifene/
62. Abiraterone Acetate/
63. bicalutamide.mp.
64. Cyproterone/ or Cyproterone Acetate/
65. enzalutamide.mp.
66. Flutamide/
67. Medroxyprogesterone/or Medroxyprogesterone Acetate/
68. Megestrol/or Megestrol Acetate/
69. norethisterone.mp. or Norethindrone/
70. Diethylstilbestrol/
71. Ethinyl Estradiol/or ethinylestradiol.mp.
72. Idarubicin/
73. Methotrexate/
74. Trifluridine/
75. lenvatinib.mp.
76. venetoclax.mp.
77. ixazomib.mp.
78. Palbociclib.mp.
79. 1 or 2 or 3 or 4 or 5 or 6 or 7 or 8 or 9 or 10 or 11 or 12 or 13 or 14 or 15 or 16 or 17 or 18 or 19 or 20 or 21 or 22 or 23 or 24 or 25 or 26 or 27 or 28 or 29 or 30 or 31 or 32 or 33 or 34 or 35 or 36 or 37 or 38 or 39 or 40 or 41 or 42 or 43 or 44 or 45 or 46 or 47 or 48 or 49 or 50 or 51 or 52 or 53 or 54 or 55 or 56 or 57 or 58 or 59 or 60 or 61 or 62 or 63 or 64 or 65 or 66 or 67 or 68 or 69 or 70 or 71 or 72 or 73 or 74 or 75 or 76 or 77 or 78
80. antineoplastic agent/ad [Drug Administration]
81. antineoplastic agent/dt [Drug Therapy]
82. antineoplastic agent/po [Oral Drug Administration]
83. antineoplastic agent/do [Drug Dose]
84. 80 or 81 or 82 or 83
85. (reduc* or attenuat* or discontinu* or modif* or dosing or schedul* or strateg* or dosage* or intercalat* or alternat* or personali* or break or holiday* or cessation or interrup* or intermit* or escala* or prescrib* or optimis* or optimiz* or tailor* or individuali* or "non conventional*" or nonconventional* or "non standard*" or nonstandard* or "treatment free").ab,ti.
86. (reduc* or attenuat* or discontinu* or modif* or dosing or schedul* or strateg* or dosage* or intercalat* or alternat* or personali* or break or holiday* or cessation or interrup* or intermit* or escala* or prescrib* or optimis* or optimiz* or tailor* or individuali* or "non conventional*" or nonconventional* or "non standard*" or nonstandard* or "treatment free").ti.
87. 79 and 84 and 85
88. 79 and 84 and 86

**Table 3:** CINAHL search strategy

2-83: First Component (drug names): name of each of the drug (same as Medline and Embase) as MESH terms, if not as keyword (title/abstract). Total number of drugs is 77 but 4 drugs had two different spellings: mercaptopurine (6-mercaptopurine), ethinylestradiol (ethinyl estradiol), norethisterone (norethindrone), medroxyprogesterone (medroxyprogesterone acetate)
84. 2 OR 3 OR 4…..OR 21
85. 22 OR 23 OR 24 OR…OR 42
86. 43 OR 44 OR… OR 63
87. 64 OR 65….OR 83
88. MESH: "ANTINEOPLASTIC AGENTS"/ad (i.e. administration and dosage)
89. MESH: "ANTINEOPLASTIC AGENTS"/tu (i.e. therapeutic use)
90. MESH: "DRUG ADMINISTRATION SCHEDULE"/
91. 88 OR 89 OR 90
92. Second component: (reduc* OR attenuat* OR discontinu* OR modif* OR dosing OR schedul* OR strateg* OR dosage* OR intercalat* OR alternat* OR personali* OR break OR holiday* OR cessation OR interrup* OR intermit* OR escala* OR prescrib* OR optimis* OR optimiz* OR tailor* OR individuali* OR "non conventional*" OR nonconventional* OR "non standard*" OR nonstandard* OR "treatment free").ti
93. 84 AND 91 AND 92 (one quarter of results, then exported)
94. 85 AND 91 AND 92 (one quarter of results, then exported)
95. 86 AND 91 AND 92 (one quarter of results, then exported)
96. 87 AND 91 AND 92 (one quarter of results, then exported)

**Table 4:** Cochrane Registry of Controlled Trials search strategy

1. First Component (drug names):
bexarotene or busulfan or capecitabine or chlorambucil or cyclophosphamide or estramustine or etoposide or hydroxycarbamide or melphalan or mercaptopurine or lomustine or mitotane or procarbazine or tegafur or temozolomide or tioguanine or topotecan or treosulfan or tretinoin or trifluridine or vinorelbine or afatinib or axitinib or bosutinib or cabozantinib or cobimetinib or crizotinib or ceritinib or dabrafenib or dasatinib or erlotinib or everolimus or gefitinib or ibrutinib or idelalisib or imatinib or lapatinib or nilotinib or nintedanib or olaparib or osimertinib or panobinostat or pazopanib or ponatinib or regorafenib or ruxolitinib or sorafenib or sunitinib or temsirolimus or trametinib or vandetanib or vemurafenib or vismodegib or thalidomide or lenalidomide or pomalidomide or anastrozole or exemestane or letrozole or tamoxifen or toremifene or abiraterone or bicalutamide or cyproterone or enzalutamide or flutamide or medroxyprogesterone or megestrol or norethisterone or diethylstilbestrol or ethinylestradiol or idarubicin or methotrexate or trifluridine or lenvatinib or palbociclib or venetoclax or Ixazomib in Title, Abstract, Keywords
2. Second component:
reduc* or attenuat* or discontinu* or modif* or dosing or schedul* or strateg* or dosage* or intercalat* or alternat* or personali* or break or holiday* or cessation or interrup* or intermit* or escala* or prescrib* or optimis* or optimiz* or tailor* or individuali* or "non conventional*" or nonconventional* or "non standard*" or nonstandard* or "treatment free" in Record Title
3. "DRUG ADMINISTRATION" or "ANTINEOPLASTIC AGENT*" in Title, Abstract, Keywords (word variations have been searched)
4. 1 AND 2 AND 3

### Data management

Three researchers will independently review search results and all duplicates will be removed. All retrieved titles will be screened against inclusion and exclusion criteria by the primary author; a quarter will be independently assessed by another member of the research team. Sources that do not meet all eligibility criteria will be excluded. Full text publications of all remaining sources will be obtained and screened against eligibility criteria by the primary author. Another member of the research team will independently screen all full text publications against eligibility criteria. Exclusion decisions of full text publications will be agreed between the research team after independent review. The number of excluded sources and reasons for exclusion will be reported. Publications reporting studies that meet all inclusion criteria will all be included in the review.

### Search expansion

An expanded search will be conducted to ensure comprehensive review evidence that meets inclusion criteria. Prospective citation chaining will be conducted in the database Web of Science to identify potentially relevant papers that have cited included studies. Retrospective snowballing of reference lists of included studies will also be conducted. Sources identified during the search expansion stage of the literature search will undergo the screening process described above. Search and exclusion process will be reported in accordance with PRISMA guidelines [9].

### Critical appraisal

Critical appraisal will be independently conducted by at least two members of the research team to identify potential bias, strengths and limitations of included studies. Standardised critical appraisal tools will be used to appraise the quality of studies; the tools used will depend on the type of study design being reviewed, but will include use of Critical Appraisal Skills Programme (CASP) tools. The following aspects of the study will be critiqued: soundness of study (method, quality of data analysis and interpretation), appropriateness of study design to answer the question (study type, sample characteristics, allocations of oral SACT prescribed at non-conventional doses, relevancy to current prescribing practice), and quality of reported outcomes over different time periods. The researchers will assign a quality rating of high, medium and low to studies reviewed and any discrepancy in quality appraisal rating will be discussed with and resolved within the research team.

### Data extraction

Members of the research team will independently extract data using pre-defined categories into data extraction tables, in order to summarise key findings for reporting. Data extraction table is available in Table 5.Since the range of published data on the topic is not fully known to the research team until evidence has been systematically identified, the process of data extraction will require an inductive approach, whereby key findings will be extracted from a range of study designs reporting a range of study outcomes. Data extraction tables will be compared and integrated to ensure all key findings are included and accurately reported in the review. Any disagreements during this process will be resolved through discussion within the research team.

**Table 5:** Data extraction table

Data to be extracted	Item
Publication ID	• Author • Publication date
Study aim	• Title/Purpose/Aim
Study design	• Study type: meta-analysis, late phase clinical trial, cohort study, cross-sectional study, retrospective study, observational study, Case-control study, case-report • Measurement tools, instruments, measures, outcome criteria
Non-conventional dosing characteristics	• Oral SACT name • Dose • Duration of therapy
Sample characteristics	• Number of participants • Country • Age • Gender • Cancer type
Findings	• Reported efficacy outcomes • Reported side effects/toxicity outcomes • Reported health-related quality of life • Any other findings
Strengths and limitations	• Findings of critical appraisal

### Data analysis and synthesis

Key findings (sorted and summarised during the data extraction process) will be integrated and a narrative summary/synthesis of findings will be presented and discussed. If sufficient stratified data is available about the population (e.g. age group, tumour type, disease stage), or intervention (drug, dosing schedule) or outcome (toxicity, adherence, QoL), a sub-group analysis will be conducted to inform prescribing practice. Results will be discussed in reference to the strengths and weaknesses of the evidence base, and with a focus on addressing the primary aim to identify evidence about non-conventional dosing of oral SACT in oncology and malignant haematology.

## Discussion

In clinical practice, non-conventional dosing of oral anticancer drugs is becoming more common in response to treatment toxicity and impaired adherence [4]. This approach enables prescribers to tailor prescribing decisions for individual patients based on their needs [11]. The main aim of this review is to identify and collate evidence about the use of non-standard dosing of oral SACT in oncology and malignant haematology. In summarising evidence, the strengths and limitations of the evidence base will be identified and discussed. A summary of key findings will be used to inform prescribers working in oncology and haematology. It will also identify any evidence of changes in toxicity and/or quality of life outcomes for patients receiving non-standard doses of oral SACT. Findings of this review will also inform a future feasibility or interventional study evaluating non-conventional dosing of oral SACT. Limitations in the evidence base may arise from variability in both the type and quality of studies, which may restrict the generalisability of conclusions about the use of non-conventional dosing for oral SACT in oncology and malignant haematology.

In addition to disseminating results of this review in an international health care journal, findings will be presented to oncologists, nurses, pharmacists and patient representatives within a local Cancer Alliance and at national and international conferences.

## Abbreviations

BNF: British National Formulary
CASP: Critical Appraisal Skills Programme
CINAHL: Cumulative Index to Nursing and Allied Health Literature
EORTC: European Organisation for Research and Treatment of Cancer
IM: Intramuscular
IMWG: International Myeloma Working Group
IT: Intrathecal
IV: Intravenous
MESH: Medical Subject Heading
MHRA: Medicines and Healthcare Products Regulatory Agency
PRISMA-P: Preferred Reporting Items for Systematic Review and Meta-Analysis Protocols
QLQ: Quality of Life Questionnaire
QoL: Quality of life
SACT: Systemic anticancer therapy
SC: Subcutaneous
